# Editorial: Translational potential of novel biomarkers and molecular targets in lifestyle disorders

**DOI:** 10.3389/fphar.2026.1768318

**Published:** 2026-03-11

**Authors:** Atin Kalra, Pradip Nirbhavane, Chhanda Charan Danta, Priyanka Saroj, Ravinder Nayak

**Affiliations:** 1 Amity Institute of Pharmacy, Amity University Haryana, Gurgaon, India; 2 Center for Translational Science, Florida International University, Miami, FL, United States; 3 Amity Institute of Pharmacy, Amity University Uttar Pradesh, Noida, Uttar Pradesh, India; 4 Postdoctoral Research Fellow at the Centre for Addiction and Mental Health (CAMH), Toronto, ON, Canada

**Keywords:** biomarker, cancer, diabetes, noncommunicable chronic disease, repurposable drugs

## Aims and scopes of this research topic

Lifestyle disorders, also known as non-communicable diseases, are chronic in nature and can be attributed to poor diet, lack of physical activity, prolonged stress, predisposition to diseases, etc. These factors thus make one vulnerable to conditions such as cardiovascular diseases, diabetes, cancer, and chronic respiratory diseases.

As per the World Health Organization (WHO), 43 million deaths have been reported due to these non-communicable diseases. In 2021. Over 18 million of these deaths were considered “premature” (before age 70), and 82% of these premature deaths occurred in low- and middle-income countries (World Health Organization, 2025).

There has been a concerted effort to address the rise of lifestyle disorders by developing better therapeutics and focusing on preventive measures through the development of newer and more efficient biomarkers.

A biomarker is defined as a substance, structure, or process that can be objectively quantified as an indicator of typical biological functions, disease processes, or biological reactions to exposure (Aryutova et al., 2021). Thus, they play a critical role in the early detection and diagnosis of these lifestyle diseases.

**FIGURE 1:**
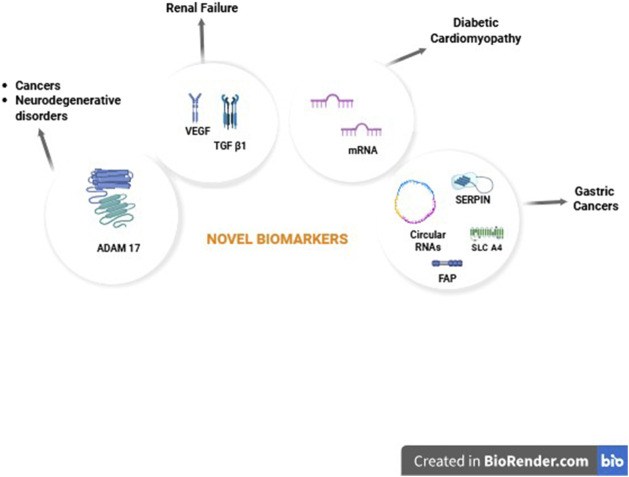
Novel biomarkers and their role in various diseases (Created in https://BioRender.com).

This Research Topic contains six articles: three reviews, two original research articles, and one clinical trial. These articles focus on the current state of the art in the area of biomarkers in various inflammatory like gastric cancers and cardiovascular diseases. Further, the role of transmembrane proteases in the pathogenesis of neurodegenerative diseases is also discussed (Figure 1).

## Shifting paradigms in biomarker research and its potential impact on treatment outcomes

### Dysregulation of a disintegrin and metalloproteinase (ADAM)

ADAM17 is a type I transmembrane protein consisting of 824 amino acids, encoded by a gene located on chromosome 2 (Rose-John, 2013). Strong evidence suggests dysregulation of ADAM17 activity has been implicated in various diseases, including inflammatory disorders, cancer, cardiovascular disease, and neurodegenerative disease (Liu et al.). Its ability to process tumor necrosis factor-alpha (TNF-α) and epidermal growth factor receptor (EGFR) ligands makes it a critical player in disease progression and a promising therapeutic target. Recent literature suggests it has a significant role in the progression of various cancers, including colorectal and gastric cancers (Chen et al., 2024). Also, its involvement in cardiovascular diseases like myocardial infarction is being investigated (Kawai et al., 2021).

### Novel biomarkers in the detection of gastric cancer (GC)

GC ranks fourth in the world for mortality among malignancies and fifth for incidence (Mamun et al., 2024).

Conventional serum markers often fail to detect GC at curable stages or distinguish it from benign conditions, resulting in missed opportunities for timely intervention. Markers used in GC, such as carcinoembryonic antigen (CEA), cancer antigen (CA19-9), and CA72-4, are constrained by low sensitivity and specificity, particularly for early-stage disease (Kaur et al.). Recent studies have identified upregulation of enzymes such as fatty acid synthase (FASN), and transcription factors like SREBP1 have been reported; both are emerging as tissue-based biomarkers associated with tumor progression and poor prognosis. Other important biomarkers include the role of circular RNAs and Proteinase 3 (PRTN3) in GC (Kaur et al.).

Similarly, microRNAs (mRNAs) have also emerged as key regulators in various diseases, including cardiomyopathy (De et al.).

### Mitochondrial dysfunction in type 2 diabetes mellitus (T2DM)

T2DM is a metabolic disorder characterised by hyperglycemia and insulin resistance. Mitochondrial dysfunction has been shown to contribute significantly to the various inflammation responses associated with T2DM. Imeglimin, an oral antidiabetic agent, has demonstrated dual-purpose action to control hyperglycemia. However, real-life evidence is limited. Satheesan et al. studied the effect of Imeglimin monotherapy and compared it with its combination therapy with other antidiabetics and assessed their impact on mitochondrial distress via circulating cell-free mitochondrial DNA (ccf-mtDNA).

### Emerging role of vascular endothelial growth factor (VEGF) and endothelin-1 (ET-1)

End-stage renal failure patients rely on frequent dialysis, which is cumbersome for both patient and caregiver, requiring advanced facilities that are often a big constraint in resource-poor countries. Current focus area to address this includes studying the role of biomarkers such as transforming growth factor-β1 (TGF-β1), VEGF, and ET-1, and their levels in peritoneal dialysis effluent on peritoneal solute transport function (Han et al.).

### Drug repurposing for managing lifestyle disorders

Recently, drug repurposing has become an important arsenal in managing various diseases like cancers. The role of ondansetron’s anti-inflammatory and antioxidant activity has been studied for possible use in reducing pro-inflammatory markers and oxidative stress in the liver (Naeem et al.). This has provided an opportunity for further investigation into its role in the management of diabetes and non-alcoholic fatty liver disease (NAFLD).

## Concluding remarks

This Research Topic has provided recent developments in the area of biomarker research and novel targets for lifestyle diseases. The emerging evidence of the role of these biomarkers in the treatment of various inflammatory diseases is likely to transform into robust clinical markers in the near future. Because of their high sensitivity, biomarkers like ADAM17, SREBP1, and circular RNA have the potential to revolutionise the early detection of GC. Similarly, other biomarkers like VEGF and ET-1 have been strongly associated with renal failure. Finally, with robust experimental evidence and proof of concept studies, these biomarkers are providing an opportunity for the repurposing of drugs like ondansetron in the management of diabetes and NAFLD.
